# Nucleophagy: from homeostasis to disease

**DOI:** 10.1038/s41418-018-0266-5

**Published:** 2019-01-15

**Authors:** Margarita-Elena Papandreou, Nektarios Tavernarakis

**Affiliations:** 1 Institute of Molecular Biology and Biotechnology, Foundation for Research and Technology-Hellas, 71110 Heraklion, Crete Greece; 2 Department of Basic Sciences, School of Medicine, University of Crete, 71110 Heraklion, Crete Greece

**Keywords:** Macroautophagy, Neurological disorders

## Abstract

Nuclear abnormalities are prominent in degenerative disease and progeria syndromes. Selective autophagy of organelles is instrumental in maintaining cell homeostasis and prevention of premature ageing. Although the nucleus is the control centre of the cell by safeguarding our genetic material and controlling gene expression, little is known in relation to nuclear autophagy. Here we present recent discoveries in nuclear recycling, namely nucleophagy in physiology in yeast and nucleophagic events that occur in pathological conditions in mammals. The selective nature of degrading nuclear envelope components, DNA, RNA and nucleoli is highlighted. Potential effects of perturbed nucleophagy in senescence and longevity are examined. Moreover, the open questions that remain to be explored are discussed concerning the conditions, receptors and substrates in homeostatic nucleophagy.

## Graphical abstract

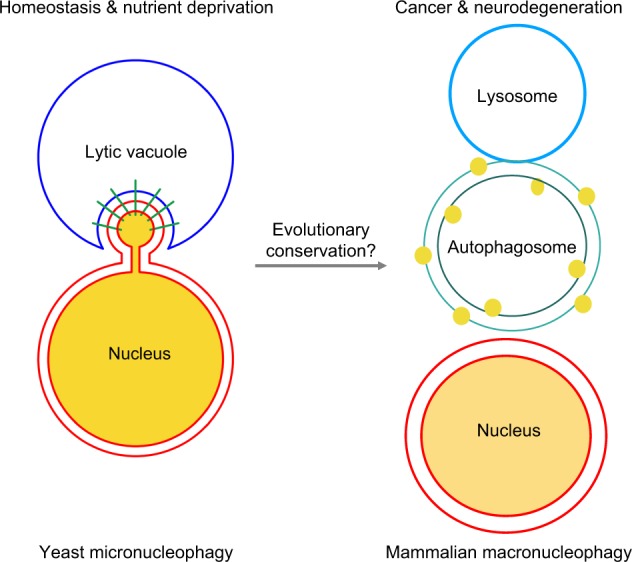

**Facts**:

- Selective autophagy of nuclear components is termed nucleophagy
- Nucleophagy is conserved from yeast to mammals
- In yeast, micronucleophagy occurs under basal and nutrient-deprived conditions
- In mammals, macronucleophagic events have been described in cancer and neurodegeneration
- Different components of the nucleus from the nuclear membrane to nucleolar components can be recycled

**Open Questions**:

- Under which physiological conditions does macronucleophagy occur?
- What are the molecular mechanisms/pathways regulating macronucleophagy under homeostasis?
- Are there different types of macronucleophagy depending on the underlying triggering conditions and different mechanisms that would selectively degrade different nuclear components?
- Does recycling of different nuclear components such as DNA, RNA and nucleoli contribute to longevity?
- Would drug targeting nucleophagy delay premature ageing?

## Introduction: General and selective autophagy

**Fig. 1:**
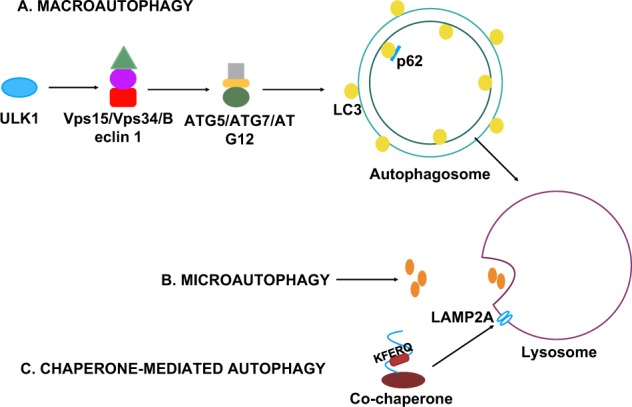
Types of autophagy. Macroautophagy involves the formation of the autophagosome. Initiation occurs with Unc-51-like kinase 1 activation and concomitant triggering of the phosphatidyl-inositol-3-kinase complex, Vps15/Vps34/Beclin 1 complex. In turn, two ubiquitin conjugation systems, ATG5/ATG7/ATG12 and LC3, are required for autophagosome formation and maturation. Autophagosome–lysosome fusion allows for degradation of autophagic substrates, such as p62. Microautophagy happens with direct interaction of the substrate and the lysosome. Chaperone-mediated autophagy requires chaperone targeting of specific proteins with the pentapeptide motif KFERQ to the lysosome-associated membrane protein LAMP-2A

Autophagy from the Greek words ‘auto’, self, and ‘phagy’, eating, is a physiological catabolic process that occurs in all eukaryotic cells to recycle defective organelles or protein aggregates [1]. Although thought to be a bulk degradation pathway, autophagy is a highly selective cellular clearance mechanism. There are three major types of autophagy, macroautophagy, microautophagy and chaperone-mediated autophagy (Fig. 1). In macroautophagy, thereby referred to as autophagy, a double-membrane vesicle called the autophagosome is formed that contains the substrates to be degraded in the lytic organelle, the lysosome, by the hydrolytic enzymes. In microautophagy, part of the organelle to be degraded pinches off and directly interacts with the lytic organelle or the lysosome; pexophagy, the selective degradation of peroxisomes [2], is an example of microautophagy [3]. In chaperone-mediated autophagy, the material to be degraded is selectively recognized by cytosolic chaperones and directed to a receptor at the lysosomal membrane (Fig. 1) [4].

Autophagy is a tightly controlled stepwise mechanism. It takes place at basal levels under physiological conditions but can be induced by several cellular stresses, such as nutrient deprivation, oxidative stress and DNA damage. Initiation occurs with nucleation sites, omegasomes, which recruit different autophagic factors and form the phagophore with the aid of the Unc-51-like kinase 1 (ULK1) complex and the class III phosphatidyl-inositol-3-kinase complex, Vps15, Vps34 and Beclin 1 [4]. Next, two ubiquitin conjugation systems, the microtubule-associated protein1 light chain 3 (LC3) and autophagy related (Atg) Atg5–Atg7–Atg12 proteins, are required for autophagosome formation and maturation that is then transported and degraded in the lysosome. Selective autophagy requires additional autophagy receptors such as p62 and NDP52, which contain an LC3 interacting region recognized by LC3B localized to the outer autophagosomal membrane [5, 6]. Mitochondria and defective proteins are usually recycled in this manner.

## Autophagy in ageing and age-related disease

Autophagy has been shown to be a mediator of youth and longevity. Both general autophagy as well as selective types of autophagy have been shown to promote lifespan extension [7]. Yeast transcriptome comparison between short- and long-lived mutants highlights the importance of autophagy in normal lifespan, while its absence perturbs amino acid deprivation-mediated lifespan extension [8]. Similarly, *Caenorhabditis elegans* autophagy mutants for *unc-51*, *bec-1*, *atg-7*, *atg-12* and *atg-18* display shortened lifespan [9, 10]. Autophagy gene expression is reduced in neurons of old *Drosophila melanogaster*, while mutational loss of *Atg1* and *Atg8* decreases longevity [7, 11]. Conversely, overexpression of *Atg1* and *Atg8* promotes lifespan extension. Importantly, the transcription factor TFEB orthologue HLH-30, a master regulator of autophagy, has been shown to extend lifespan in the nematode [12]. In mice, overexpression of Atg5 increases autophagy and concomitantly lifespan [13]. Transcriptional downregulation of *Atg5*, *Atg7* and *Beclin-1* is prominent in the brains of aged humans [14, 15]. Aside from core autophagic proteins influencing lifespan, there are cell signalling pathways that fine tune autophagy levels and determine longevity. Lifespan extension by low insulin signalling in *C. elegans daf-2* mutants is perturbed upon autophagy inhibition [16]. Overexpression of the transcription factor FOXO in the muscles of the fruit fly promote proteostasis and anti-ageing effects through autophagy [17]. Similarly, screening in centenarians has revealed a strong correlation between longevity and insulin-like growth factor I mutations [18]. Genetic or pharmacological inhibition of the mammalian target of rapamycin (mTOR) pathway by rapamycin, which mimics dietary restriction and reduces protein synthesis while accelerating catabolic processes such as autophagy, has been shown to extend lifespan in yeast, nematodes, fruits flies and mice [19–23]. Another substance found in fruit and vegetables, resveratrol, induces Beclin-1-independent autophagy and SIRT1 activation leading to longevity [24, 25]. Spermidine has also been shown to promote lifespan extension from yeast to humans via autophagy activation [26]. Importantly, dietary restriction has been shown to induce longevity and health span in all species tested and most importantly in humans [7, 27]. Autophagy is essential for dietary restriction-mediated longevity [28].

Perturbation of autophagy is also apparent in age-related disease. In particular, in the liver, ULK1, Beclin-1 and LC3 are reduced in osteoarthritis [29]. Moreover, there are age-dependent alterations in autophagic degradation in the liver [30]. Tissue-specific autophagy gene knockout mice have been used instead of traditional knockouts that have developmental defects and postnatal lethality [15]. Conditional knockouts mimic age-related phenotypes. In the central nervous system, *Atg7* knockout neurons display ubiquitin-positive inclusion bodies, uncoordinated movement and premature death [31]. In neurodegenerative mouse models of diseases, such as Huntington or Alzheimer, in which huntingtin or amyloid-beta protein aggregates form, respectively, the pharmacological activation of autophagy ameliorates disease symptoms [32, 33]. Of note, inducing autophagy in certain age-related neurodegenerative disorders can have adverse effects on disease progression, because albeit the apparent increase on autophagosomes, there is a defect in autophagosome–lysosome fusion, thus degradation. Similarly, *Atg7* ablation causes muscle atrophy, reduction in strength and reduced differentiation of myogenic progenitors into brown adipose tissue leading to sarcopenia [26]). Interestingly enough, overnutrition can produce similar consequences leading to sarcopenia and metabolic defects [34].

Apart from general autophagic factors affecting the rate of ageing, selective micro- and macro-autophagy are critically important for catabolic processes that produce key metabolites, such as autophagy-derived amino acids, needed to maintain and increase anabolic processes, including protein synthesis. Ultimately, autophagy contributes to sustain metabolic homeostasis. Defective mitochondrial clearance can cause mitochondrial reactive oxygen species accumulation, mitochondrial DNA damage, defective mitochondrial function, impaired stress resistance and decline in cellular function such as in the case of neurodegeneration [35]. Macrolipophagy in response to nutrient deprivation can also halt lipid droplet storage, metabolic disease and age-dependent lipid deposition [36]. p62 is an autophagic receptor and substrate itself regulating selective autophagy such as mitochondrial and ubiquitinated protein recycling. Its absence triggers mature-onset obesity, leptin resistance, glucose and insulin intolerance highlighting its importance in metabolic health [37].

## Nucleophagy in yeast

Selective autophagy is essential for organismal homeostasis. The nucleus is the largest organelle in eukaryotic cells and contains a variety of different constituents that are critical for the cellular health and inheritance to the next generation. The genetic material, DNA, the mRNA, ribosomal RNA (rRNA) and the nucleolus—the site of ribosome biogenesis—are surrounded by the nuclear envelope that includes a multitude of proteins. The renewal and recycling of the nucleus has drawn growing attention. Digestion of nuclear components in yeast *Saccharomyces cerevisiae* has been shown to occur via microautophagy, in the absence of autophagosome formation, either by piecemeal nucleophagy (PMN) or late nucleophagy (LN). mTOR inactivation and subsequent nuclear envelope morphology protein/sporulation 7/phosphatidic acid phosphohydrolase axis activation triggered by nutrient deprivation, nitrogen starvation or rapamycin treatment is crucial for both micronucleophagy and macronucleophagy [38]. The main characteristics of these physiological cellular processes are summarized in Table 1.

**Table 1:** Comparison of major features of piecemeal nucleophagy (PMN) and late nucleophagy (LN) in yeast

Yeast nucleophagy characteristics and factors	PMN	LN
Macroautophagy genes	*atg1*, *atg2*, *atg3*, *atg4*, *atg5*, *atg7*, *atg8*, *atg9*, *atg13*, *atg18*, *atg17*, *atg29*, *atg31*	*atg1*, *atg2*, *atg3*, *atg4*, *atg5*, *atg7*, *atg8*, *atg9*, *atg10*, *atg12*, *atg13*, *atg16*, *atg18*, *atg23*, *atg29*, *atg31*
Cvt-specific genes	*atg11*, *atg20*, *atg24*	No
Receptor-like function	Nvj1p, Vac8p	Atg39
Inducer	Nutrient rich and early nitrogen starvation/mTOR inactivation	Prolonged nitrogen starvation/mTOR inactivation
Pathway	TORC1 inactivation, Nem1/Spo7-Pah1 axis	TORC1 inactivation, Nem1/Spo7-Pah1 axis
Nuclear shape	Unaltered	Irregular
Nature of nuclear-derived puncta	More stable	Less stable
Vps34 PtdIns(3)P-kinase complex I	Yes	No
Nuclear–vacuole junctions	Yes	No

*mTOR* mammalian target of rapamycin

**Fig. 2:**
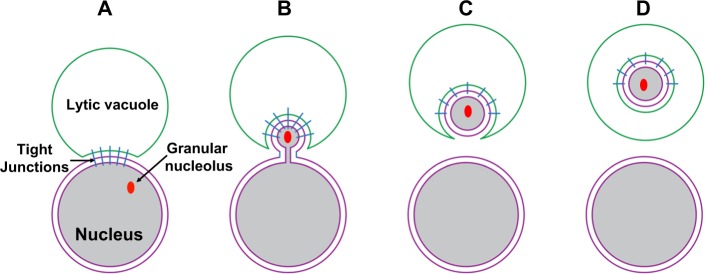
Piecemeal nucleophagy in yeast. Micronucleophagy occurs both under nutrient-rich conditions as well as nitrogen deprivation. The nucleus (purple) and the lytic vacuole (green) directly interact via tight junctions (blue) (**a**). In turn, nuclear ER bulges form **b** containing nucleophagic substrates such as the granular nucleolus (red), which are then pinched off (**c**). Finally, the nuclear derived vesicles are degraded in the lytic vacuole (**d**)

PMN takes place under nutrient-rich conditions as well as after a short period of nitrogen starvation. This process initially requires direct contact between the cell nucleus and degradative organelle, the lytic vacuole. Soon after, nucleus–vacuole junctions are established, a step that has been shown to be independent of autophagic proteins (Fig. 2a) [39]. Subsequently, outer nuclear membrane and nuclear endoplasmic reticulum (ER) protrusions form, which then develop into nuclear ER-derived vesicles surrounded by the vacuole (Fig. 2b). In turn, these vesicles are completely pinched off the nucleus (Fig. 2c). Ultimately, fusion of the membranes at multiple points allows PMN release into the vacuole to be subsequently degraded by vacuolar hydrolases (Fig. 2d). Outer nuclear membrane nucleus to vacuole protein 1 (Nvj1p) and vacuolar protein 8 membrane are essential components of PMN. Substrates of PMN include nuclear envelope components, the granular nucleolus containing pre-ribosomes excluding nuclear pore complexes and spindle pole bodies [40]. Recently, RNA non-selective bulk degradation has been detected after nitrogen starvation [41].

Of note, tethered nuclear blebs or PMN vesicles (micronuclei) are formed in the absence of certain core autophagy genes such as Atg7, Atg8, Atg9 and Atg18 [39]. However, PMN cannot be completed in their absence as the final vacuolar membrane fusion step is defective. Generally, this bona fide microautophagic process requires a complex of general autophagy factors of the Ub-like conjugation system, the Atg9 cycling system, macroautophagy-specific proteins and lipid biosynthesis components. Additionally, Atg11 and partially Atg20 and Atg24 proteins of the cytoplasm-to-vacuole pathway, a yeast-specific specialized form of microautophagy active in nutrient-rich conditions, are also involved.

In contrast to PMN, LN is only induced after prolonged periods of nitrogen starvation (18–24 h) and causes nuclear shape alterations. Apart from being temporally differentiated to PMN, it occurs in cells that do not almost invariably undergo PMN at the same time. The temporal and spatial distinction between the two nucleophagic processes has been shown using differential reporters, namely a nuclear membrane reporter Nvj1p-EYFP for the former, and a nucleoplasm reporter, NAB35-DsRed, in combination with a nuclear Rosella, n-Rosella, which is a pH biosensor, for the latter [42, 43]. Distinctively, essential macroautophagic genes such as *atg1* and *atg8* cause efficient hindering of LN. This leads to deformed nuclei implying excessive accumulation of nuclear components that fail to be degraded.

More recently, a form of nucleophagy has been dissected. Atg39, a receptor for yeast nucleophagy located at the perinuclear ER, has been described. This protein is dispensable for yeast micronucleophagy, PMN, but is required for efficient ER and nuclear component recycling [44]. In particular, autophagic substrates of Atg39-dependent nucleophagy have been identified. Outer and inner nuclear membrane proteins Hmg1 and Src1 as well as nucleolar protein Nop1 autophagic degradation indicate that Atg39 is a master regulator of different nuclear constituents. Interestingly enough, Atg39 protein levels increase with rapamycin treatment which implies that it is a receptor but not a substrate for nucleophagy [44]. Whether this mechanism is evolutionarily conserved in mammals remains to be determined as no Atg39 homologue has been identified. CLIP and cohibin are critical in promoting micronucleophagy in yeast by promoting nucleolar protein degradation while excluding rDNA. This is accomplished by relocalizing the former to sites proximal to nuclear–vacuolar junctions and tethering the latter to the inner nuclear membrane [45]. All in all, this process is essential for maintaining yeast nuclear shape integrity and ultimately cell viability under excessive nutrient, nitrogen, deprivation. However, whether these nuclear/nuclear ER-derived vesicles are separate from autophagosomes remains to be determined.

## Macronucleophagy in the pathophysiology of higher organisms

The interplay between nuclear dynamics and the macroautophagic machinery has not been extensively studied [46]. However, it has been shown that multiple autophagosomal proteins are localized to the nucleus. Acetylation of LC3 causes its nuclear localization while its deacetylation during starvation triggers its transport from nucleus to the cytoplasm. Whether, under homeostasis, LC3 has a physiological role in the nucleus apart from storage purposes has yet to be determined [47]. ATG5 and ATG7 have also been identified in the nucleus regulating p53 activation, cell cycle arrest and cell death concurrently to autophagy [48, 49]. P62 continuously shuttles between the nucleus and the cytoplasm. In the nucleus, along with autophagic adaptor ALFY, P62 acts synergistically to transport misfolded ubiquitinated proteins to the promyeolocytic leukaemia (PML) protein bodies. Upon stress, ALFY is transported to the cytoplasm and colocalizes with cytoplasmic p62 and ubiquitin-positive structures [50].

Examples of macroautophagic recycling of nuclear components in physiology that have been described for developmental purposes are fungi for organismal proliferation and epidermal keratinocytes for cell differentiation. Moreover, there is growing substantial evidence underlying the global significance of nucleophagy in organismal homeostasis. When perturbed, diverse pathologies can occur, for example, neurodegeneration and cancer.

An interesting example of macroautophagic degradation of whole nuclei is that of the filamentous fungus *Aspergillus oryzae*. This organism consists of hyphae, which contain multinucleate cells [51]. Particularly, basal cells can simultaneously recycle nuclei, mitochondria and peroxisomes. Whether autophagosomes encircling whole nuclei are distinct from other selective autophagy autophagosomes has not been addressed. However, these autophagosomes are large and could be specific for nuclei. Not only does whole nucleus recycling not kill the cell, as it has other nuclei as well, but instead the degradation products contribute to the proliferation at the hyphal tips of the organism by nutrient recycling. Thus macroautophagic nucleophagy in this type of fungus acts as a nutrient storage source for continuous proliferation [46].

Moreover, renewal of the mammalian epidermis occurs by a basal layer of proliferating keratinocytes, which after a few divisions undergo epidermal terminal differentiation into corneocytes. During this process, it is suggested that nucleophagic events occur both in mouse and human cells [52]. Indicative is the fact that perinuclear LC3B-decorated vesicles contain the autophagic receptor and substrate p62, heterochromatin protein 1α, lamin A and B1. In psoriasis, a chronic, immune-mediated, inflammatory skin disease, there is lack of autophagic markers in the psoriatic skin lesions. However, no experimental evidence can support psoriasis being an outcome of defective nucleophagic events.

**Fig. 3:**
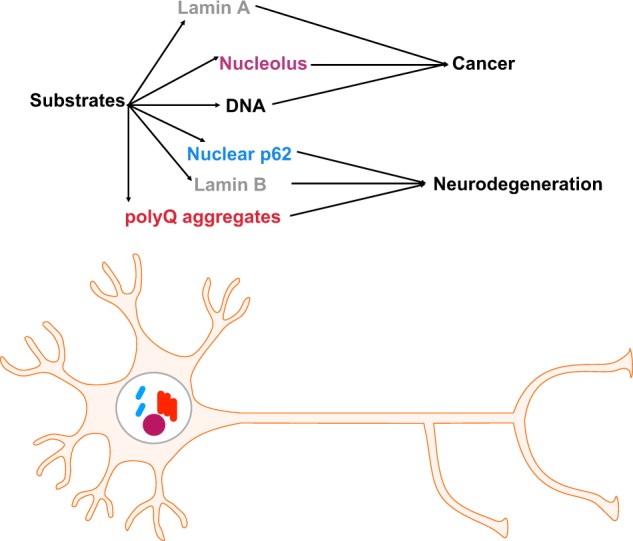
Macronucleophagy in disease. Multiple autophagy substrates have been identified in pathological settings. In cancer, lamin A and B (grey) as well as nucleolar components (purple) have been identified while DNA (black) when not degraded properly could cause oncogenic stress. Neurodegeneration can occur as a result of lack of functional nuclear protein clearance, nucleophagy, of PolyQ aggregates (red) or of nuclear autophagy receptor p62

Accumulation of oncogenic mutations as a response to DNA damage in cells transform them into cancerous. Among the alterations observed, the nuclear architecture is perturbed. Nuclear shape changes occur together with nuclear matrix protein composition [53]. This is accompanied by chromosomal territories and PML body relocalization. Importantly, the largest structure of the nucleus, the nucleolus, is usually enlarged with functional consequences concerning ribosome biogenesis leading to increased protein synthesis [54]. Autophagy is essential after oncogenic insults caused by DNA-damaging agents such as etoposide pro-oxidant hydrogen peroxide to induce cellular senescence [55]. More recently, lamin B has been identified as an autophagic substrate after oncogenic hyperactivation. Specifically, activated KRAS in primary human cells induces the direct interaction of nuclear LC3 and lamin B1 via a direct LC3 interacting region (LIR) domain. LC3 together with lamin B1 and heterochromatin domains are then transported to the cytoplasm for autophagic degradation [56]. Importantly, mTOR inhibition by starvation or rapamycin treatment does not trigger LC3-dependent lamin B degradation highlighting the selective nature of nucleophagy. Thus oncogenic stress leads to downregulation of lamin B. Depletion of ATG7 or mutation of the LIR domain of lamin B leads to halt in lamin B degradation, concomitant cell cycle arrest and senescence. The mechanism by which nucleophagy triggers cellular senescence remains to be elucidated. Recent data have revealed that lamin B expression after DNA damage/ultraviolet irradiation in skin is a biomarker of cellular senescence, skin regeneration and therefore photoageing [57]. Nucleophagy in cancer could act as a compensatory mechanism to halt proliferation and induce senescence in the tumour cell. Thus nucleophagy could be potentially therapeutically targeted by drugs for therapeutic purposes to promote tumour suppression (Fig. 3). Another autophagic degradation pathway that has been identified and could be referred to as nucleophagy is that of damaged nuclear DNA, which can cause inflammation, autoimmunity and cancer (Fig. 3). DNA damage itself increases LC3 while extranuclear DNA is found in buds or small speckles as a result of DNA damage, autophagy or DNase2a deficiency [58]. Hence, DNA lysosomal degradation could promote cellular senescence as well.

As described above, general and selective macroautophagy has been shown to be a critical denominator for several neurodegenerative diseases. Polyglutamine (PolyQ) diseases and cerebellar disorders are a group of neurological disorders triggered by the accumulation of CAG repeats in polyQ stretches in the respective proteins [59]. Recently, activation of a newly described type of nucleophagy has been observed in Dentatorubral-pallidoluysian atrophy patients that is caused by mutant atropin, with excess CAG repeats displaying epilepsy, dementia and ataxia symptoms. Interestingly enough, mechanistically in this disease there is disturbance of canonical autophagy, which is coupled with non-canonical Golgi membrane-associated degradation of lamin B and its subsequent excretion [60, 61]. Lamin B recycling, relocalization and excretion leads to alterations in transcription and nucleocytoplasmic transport. There is great resemblance between autophagy deficiency and this alternative Golgi-dependent nucleophagy that ultimately leads to irreparable damage, nuclear collapse and neuronal degeneration (Fig. 3). Concomitantly, this nuclear breakdown causes nuclear p62 accumulation coupled with DNA damage and cellular senescence is observed.

The question that remains is whether lamin B degradation, via the autophagolysosome system as well as Golgi membrane-associated degradation and excretion, occurs in a healthy cell and whether other nuclear proteins are cleared out in the same manner. However, it is possible that dysfunctional autophagy may trigger exacerbation/hyperactivation of Golgi-dependent lamin B degradation and excretion due to polyQ accumulation, in an effort to clear out the aggregates. Ultimately, this would accentuate the requirement for maintaining nuclear integrity rejuvenation. Thus it is unclear whether this type of nucleophagy occurs only under acute stress/pathological conditions or whether it exists, albeit at lower levels, under homeostasis. Nevertheless this example accentuates the fact that, as in the case of autophagy, excess nucleophagy is detrimental for cell viability.

Inefficient clearance of nuclear aggregates in other neuronal inclusion diseases could be attributed to dysfunctional nucleophagy. Protein-mediated polyQ disorders include Huntington disease, spinocerebellar ataxias and spinobulbar muscular dystrophy caused by mutant huntingtin, ataxins and androgen receptor, respectively [62–64]. Of note, all these proteins are ubiquitinated but insufficiently degraded. Oculopharyngeal muscular dystrophy, caused by an expansion of alanine in the PABPN1 protein and RNA-mediated fragile X‐associated tremor ataxia syndrome caused by mutant FMR1 mRNA, could also be affected by disturbed nuclear recycling [65, 66]. An interesting disease is multiple muscle atrophy that is a sporadic late-onset neurodegenerative disease, the genetic link of which has not been identified. Its nuclear aggregates can be filaments associated with the nucleolus or rod-like filaments associated with the inner nuclear membrane [67].

## Nuclear structure and nucleophagy in ageing

The nucleus is the largest double membrane organelle and safeguards the cell’s genetic material, transcription and ribosome biogenesis. Different tissues exhibit dysmorphic nuclei in pathological conditions and ageing. Age-related disease and ageing are accelerated as a result of nuclear envelope or nucleoplasm protein dysfunction. In *C. elegans*, nuclear loss in the tail and intestine has been observed with apparent nuclear shrinkage and degradation [68, 69]. This is independent of apoptosis and rescued in the long-lived *daf-2* mutants. The exact mechanism by which these morphological changes occur has not been delineated although electron microscopic images indicate phagocytosis of the shrunk nuclei. It is hypothesized that nuclear recycling via autophagy could occur to provide the organism with nutrients.

There are approximately 20 different diseases caused by lamins and lamin-associated proteins ranging from accelerated ageing disorders, lipodystrophy and striated muscle diseases [70]. Ageing-related phenotypes have been attributed to nuclear envelopathies caused by outer nuclear membrane proteins. Nesprin 1 and 2 are large outer nuclear membrane proteins. Mutations in these proteins have been shown to cause enlarged nuclei and muscle age-related defects. Examples include Emery–Dreifuss muscular dystrophy with skeletal muscle wasting and heart defects, dilated cardiomyopathy and smooth muscle cell senescence [71, 72]. In the latter case, Nesprin 2 controls nuclear extracellular signal–regulated kinase 1/2 compartmentalization to PML bodies in response to DNA damage that concomitantly causes cellular senescence [73].

There is a correlation between laminopathies and dysfunctional nuclear protein clearance. Fibroblasts that have mutated lamin A or emerin display large perinuclear autophagosomes/autolysosomes and increased LC3-ll, suggesting lower autophagic flux and increased cell death [74]. Moreover, prelamin A maturation to lamin A requires farnesylation, endoproteolysis and carboxymethylation. In Hutchinson–Gilford progeria syndrome, progerin, a prelamin A form that contains the prenylated carboxyl-terminal moiety, accumulates and remains permanently attached to the nuclear envelope. This causes nuclear deformation, function perturbation and nucleophagic degradation of progerin and other nuclear components [75]. Rapamycin could be a therapeutic approach to induce progerin autophagic degradation as it increases the expression of prelamin A endoprotease ZMPSTE2 [76]. This goes in line with life-extending effects of rapamycin in genetically heterogeneous mice fed with rapamycin late in life [22].

The nucleolus has recently been identified as an ageing biomarker both in vitro and in vivo. In two complementary studies using *C. elegans* and cell cultures as models, nucleolar size is shown to be predictive of the age of the organism, the larger the size, the older the cell [77, 78]. Long-lived nematode strains mutant for *daf-2*, *eat-2*, *ife-2* and *glp-1* exhibit smaller nucleoli. Importantly, directly controlling the nucleolar size affects ageing. NCL-1, a protein that blocks nucleolar protein fibrillarin production involved in rRNA synthesis and maturation, promotes lifespan extension [79]. Physiologically, this can be accomplished by dietary restriction. Conversely, fibrillarin expression is directly proportional to nucleolar size leading to premature ageing. Molecular pathways that increase life expectancy have reduced fibrillarin levels and ribosomal biogenesis. The second study reveals that, in Hutchinson–Gilford progeria syndrome, cells have larger nucleoli and enhanced ribosome biogenesis leading to increased protein translation. Moreover, lamin A constrains nucleolar size, while mutant prelamin A progerin expands it.

Although in yeast there are indications of nucleolar degradation by PMN, whether nucleoli are recycled by selective macroautophagy in mammals has not been determined yet. The conditions under which nucleolar components would be degraded could be nutrient deprivation. As described above, nucleoli become smaller under caloric restriction. This could imply that, apart from hindering nucleolar expansion and ribosomal biogenesis, a catabolic process could be induced, that of nucleolar autophagy. In that case, different nucleolar constituents could be directed for recycling from fibrillar centres, dense fibrillar components and a granular component [80]. For instance, selective degradation of fibrillarin and rRNA would be performed to control ribosome biogenesis, protein synthesis rate and ultimately the ageing rate. How would this be mechanistically accomplished? LC3 has been shown to reside in the nucleus. If fibrillarin contains an LC3-interacting motif, direct interaction of fibrillarin and LC3 could occur with subsequent transport to the autophagosome in the cytoplasm to be degraded. Alternatively, a nucleophagy receptor could be present. In particular, p62 autophagic receptor and substrate and ALFY adaptor could be recruited to interact with fibrillarin in the nucleus and in turn deliver it to the autophagosome. In a third scenario, there could be a nucleophagy receptor/receptors on the outer nuclear membrane as in mitophagy that simultaneously indirectly interact with the autophagic substrate and the cytoplasmic autophagic machinery [81].

Interestingly enough, reproductively aged mice display large nucleoli and increased ribosomal number in the mammalian oocyte that could be an indicator of decreased oocyte quality and thus fertility [82]. It would be interesting to examine whether nucleophagy naturally occurs in oocytes and whether infertility is a result of disturbed nucleophagy.

## Conclusion and future perspective

All in all, recycling of the cell’s inner constituents is essential for its youthful nature. Nuclear recycling has been shown to occur in yeast via micronucleophagy in physiological and low-nutrition conditions. In nematodes, there is evidence of nuclear autophagy in intestinal cells during ageing; however, the mechanism by which it occurs has not been pinpointed. In mammals, different types of macronucleophagy have been observed in pathological settings, such as cancer and neurodegeneration. Macronucleophagy in transformed cells has been shown to trigger cellular senescence, which in turn contributes to inflammation and an ageing phenotype. Hence, it could be a matter of hyperactivation of nucleophagy leading to an ageing/degenerative phenotype while basal levels of nucleophagy could preserve cellular homeostasis [83]. It is quite intriguing to discern whether a physiological type of nucleophagy occurs in mammals. Until now, oncogenic stress and neurodegeneration have been instigators of disease-related nucleophagy. Although both types of nucleophagy share some common core autophagic players, neither during homeostasis nor under caloric restriction, as in the case of yeast, has nuclear recycling been observed. An Atg39 homologue has not been identified in mammals. Furthermore, nucleolar substrates of autophagy have not been observed in macronucleophagy in mammalian disease. Nevertheless, both in homeostatic yeast nucleophagy and pathological mammalian autophagy elements of the nuclear envelope are degraded by autophagy.

**Fig. 4:**
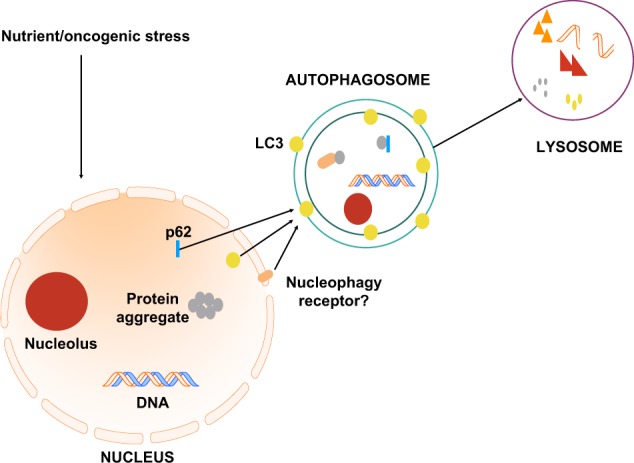
Schematic diagram of potential model of nucleophagy under homeostasis. Under nutrient/oncogenic stress, different protein, ribosomal, rRNA and DNA constituents of the nucleus are degraded via macronucleophagy with direct interaction with nuclear LC3, interaction with the autophagic receptor p62 or a specific nucleophagic receptor located at the nuclear membrane to be transported to the autophagosome. Ultimately, nucleophagic substrates are delivered into the lysosome for degradation

There are indications that recycling of the nuclear envelope is indeed faulty in progeria syndromes. Inference could be made on nuclear membrane or lamina recycling being critical at basal levels for homeostasis, while its failure could significantly contribute to ageing. An attractive model for macronucleophagy is presented in Fig. 4, where nucleophagic degradation of either nucleolar components, such as fibrillarin and rRNA, DNA or nuclear proteins is portrayed. Nuclear autophagy, which has been suggested in yeast and cancerous settings, could occur at basal levels and be induced under either nutrient or oncogenic stress to reduce ribosomal biogenesis and consequently protein synthesis or clear out protein aggregates or damaged DNA. It could require a nucleophagic receptor embedded on the outer nuclear membrane or the nucleophagic substrate could directly interact with the autophagic machinery in the nucleus. This caloric restriction-induced nucleophagy would maintain a small nucleolar size, as caloric restriction has been shown to reduce nucleolar size, and contribute to the youthfulness of the cell, ultimately of the whole organism. Moreover, it would further explain how catabolism and autophagy is perturbed during ageing while concomitantly anabolism and protein synthesis increases. Nevertheless, fine-tuning nucleophagic degradation is potentially essential to prevent age-related disease and cancer.
